# Optical Properties of Thick TiO_2_-P25 Films

**DOI:** 10.3390/nano15020099

**Published:** 2025-01-10

**Authors:** Grazia Giuseppina Politano

**Affiliations:** Department of Environmental Engineering, University of Calabria, 87036 Rende, Italy; grazia.politano@unical.it

**Keywords:** titanium dioxide, coatings, films

## Abstract

In this study, TiO_2_-P25 films on FTO substrates were synthesized using the sol-gel process and studied using Variable Angle Spectroscopy Ellipsometry (VASE) to determine their optical constants and thickness. The measurements were carried out at room temperature in the wavelength range of (300–900) nm at incident angles varying from 55° to 70°. The resulting thicknesses were found to be around 1000 nm. A graded layer model, which allowed for accurate representation of the depth-dependent optical variations, was employed to model the properties of these TiO_2_-P25 films. This modeling approach provided deeper insights into the internal structure of the films, particularly how the graded structural characteristics impact the overall optical behavior. Understanding these depth-dependent variations is essential for optimizing the use of TiO_2_-P25 films in technologies such as solar cells and optical devices.

## 1. Introduction

Titanium dioxide (TiO_2_) [1,2,3,4,5,6] has emerged as a leading semiconductor photocatalyst [7,8,9,10] due to its remarkable properties, including non-toxicity [11], chemical stability [12], high photocatalytic activity [13] and environmental compatibility [14,15]. These features make TiO_2_ particularly promising for a wide range of applications [16,17,18,19], especially as a film coating on various substrates [20]. TiO_2_ exists in three polymorphic forms [12]: anatase [21], rutile [22] and brookite [23]. Among these, rutile [24] is the most thermodynamically stable form and is commonly found in nature. Anatase [25], despite its larger bandgap of 3.2 eV [25] compared to rutile, demonstrates superior photocatalytic activity owing to its indirect bandgap, which prolongs the lifetime of photogenerated charge carriers and reduces the recombination rate of electron–hole pairs [26]. Brookite is the least common [27] and is often considered a metastable phase, transitioning between anatase and rutile.

TiO_2_ is commercially available in many forms, including pure anatase [28] and mixed-phase products like P25 [29], which is a mixture of anatase (80%) and rutile (approximately ≤20%), ideal for photocatalysis. P25 is widely recognized for its favorable electrochemical and photocatalytic properties [30], making it a cost-effective choice for numerous photocatalytic applications.

TiO_2_-P25 films [31] are highly regarded as surface coatings due to their excellent photocatalytic properties. Synthesis methods such as sol-gel processing [32], electrophoretic deposition [33] and the use of doctor blades [32] were employed to produce TiO_2_-P25 films with tailored properties suitable for several applications.

Recent research has focused on the use of TiO_2_-P25 films as photoelectrodes in solar cells [34] and as catalytic surfaces for water purification [32]. These applications have led to renewed interest in studying the optical properties of TiO_2_-P25 films [35] using ellipsometry. Variable Angle Spectroscopic Ellipsometry (VASE) [36,37,38] is particularly well-suited for the nondestructive characterization of thin films and bulk materials, offering high sensitivity and precision in measuring both optical constants and film thickness [39]. However, most of the existing literature on ellipsometry of TiO_2_ focuses on thin films, typically less than a few hundred nanometers thick, where the optical characterization is relatively straightforward [35].

Herein, TiO_2_-P25 films synthesized using the sol-gel process were studied using VASE to determine their optical constants and thickness. The focus of this study is 1000 nm thick TiO_2_-P25 films, which present unique challenges in characterization due to their substantial thickness and potential inhomogeneities. The novelty of this work lies in addressing these challenges, as VASE measurements are typically more straightforward for thinner films, making this study a significant step forward in understanding and characterizing thicker films with complex structures.

These films likely possess an inhomogeneous structure, where the optical properties vary along with the depth. This inhomogeneity makes techniques like VASE particularly challenging but also valuable for analyzing the film’s properties. The graded layer models [40] used in VASE help to provide a more detailed analysis of such films, accounting for the varying composition throughout the layers.

## 2. Materials and Methods

The TiO_2_-P25 samples used in this study were prepared using a sol-gel method [41].

Titanium dioxide (TiO_2_) nanoparticles (P25) were used to prepare a colloidal suspension (sol) by dispersing 3.2 g of the powder into 15 mL of deionized water. The mixture underwent sonication for approximately 48 h to disrupt nanoparticle aggregates and enhance dispersion. To improve stability and prevent re-aggregation, Triton X-100 (a surfactant) (Sigma Aldrich, St. Louis, MO, USA) and PEG-20000 (polyethylene glycol) (Sigma Aldrich, St. Louis, MO, USA) were added. The PEG-20000 also served to occupy interstitial spaces between the nanoparticles. The sol was sonicated further to achieve optimal uniformity. The resulting sol was deposited onto fluorine-doped tin oxide (FTO)-coated glass slides using spin coating, producing thin and uniform films. The coated substrates were then calcined at 451 °C for a minimum of 30 min to facilitate nanoparticle sintering and the removal of organic components, resulting in a porous and well-adhered TiO_2_ film. These films demonstrated stability in water, indicating strong adhesion to the FTO substrate. To achieve films with greater thickness, the deposition and sintering steps were repeated multiple times under identical conditions.

VASE was carried out to estimate both the thickness and the optical properties, n (refractive index) and k (extinction coefficient), of the samples. The WVASE32 [40] program was employed to analyze the ellipsometric data. It uses regression analysis and the Mean Squared Error (MSE) method to fit the model to the experimental data and uses the covariance matrix to provide error bars for the measured values. The optical model and parameter values for the films focus on minimizing the MSE [42]. The ellipsometric parameters, ψ and Δ, were measured using a J.A. M2000 F (Woollam Co., Lincoln, NE, USA) rotating compensator ellipsometer. This measurement covered a wavelength range of (300–900) at incident angles varying from 55° to 70° in 5° increments, all conducted at room temperature.

MSE [40] is defined by the following:

(1) $$MSE=\sqrt{\frac{1}{2N-M}\overset{N}{\underset{i=1}{\sum }}\left[{\left(\frac{\left({\psi_{i}}^{\mathrm{mod}}-{\psi_{i}}^{\exp}\right)}{{\sigma_{\psi ,i}}^{\exp}}\right)}^{2}+{\left(\frac{\left({\Delta_{i}}^{\mathrm{mod}}-{\Delta_{i}}^{\exp}\right)}{{\sigma_{\Delta ,i}}^{\exp}}\right)}^{2}\right]\;}$$

where N is the number of data points, M is the number of fitting parameters, ($\Psi^{\exp},\Delta^{\exp})$ and ($\Psi^{\mathrm{mod}},\Delta^{\mathrm{mod}})$ are the measured and modeled ellipsometric angles, respectively, and $\sigma_{\psi }$ and $\sigma_{\Delta }$ are the standard deviations of the measured ellipsometric angles. The modeled ellipsometric angles ($\Psi^{\mathrm{mod}},\Delta^{\mathrm{mod}})$ are functions of the all fit parameters that define the multilayer optical models.

**Figure 1:**
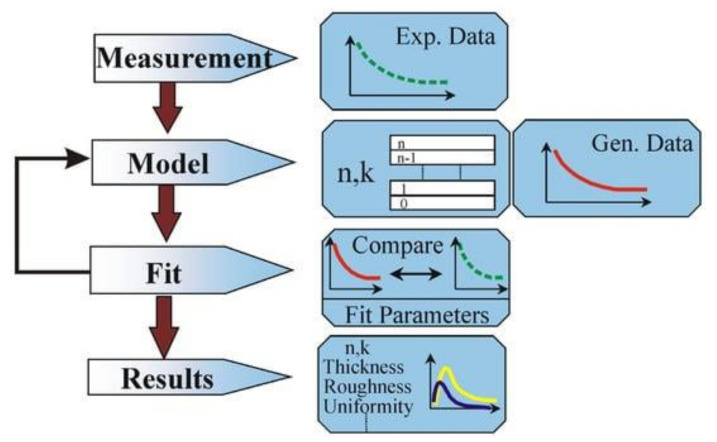
A scheme of the ellipsometry data analysis [40].

Figure 1 shows the data analysis process.

The process begins with the execution of measurements. The model is then used to calculate the predicted determinations from Fresnel’s equations, which describe each material’s thickness and optical constants. In cases where these values are unknown, an estimate is used for preliminary calculations. The generated values (red lines in Figure 1) are then compared to the experimental data (green lines) using MSE as a figure of merit to measure the fit quality. At the end of the process, we observe the resulting optical constants n and k, depicted in Figure 1 in blue and yellow lines. Further details on VASE theory and data analysis can be found in the Supplementary Materials.

## 3. Results and Discussion

**Figure 2:**
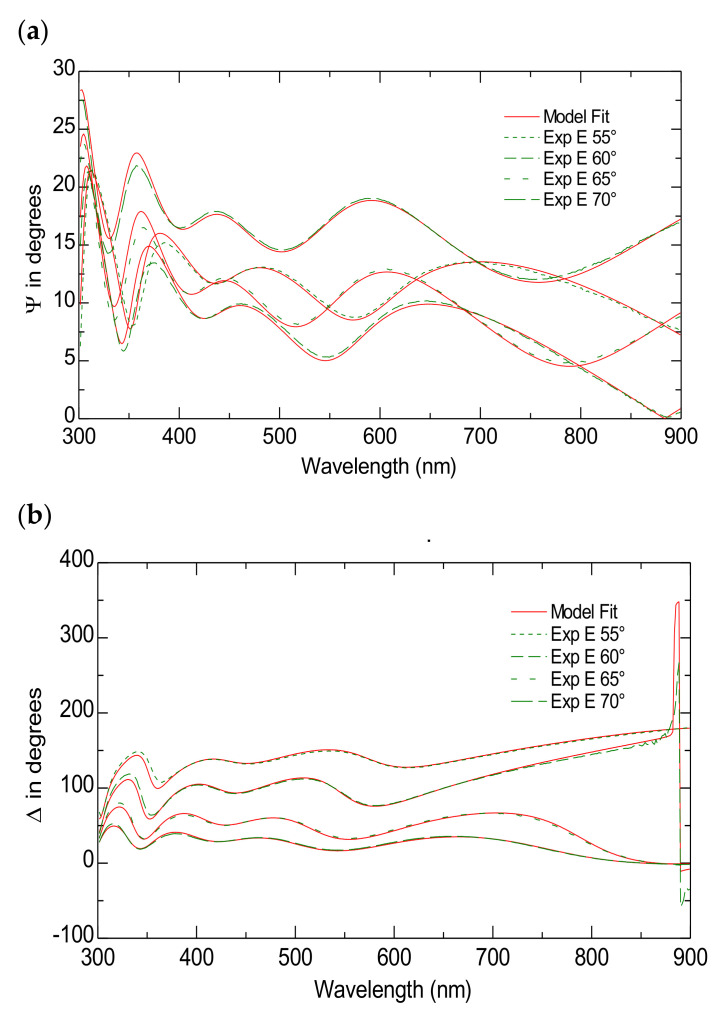
The Variable Angle Spectroscopic Ellipsometry measurements of the FTO substrate. The experimental and model-generated ψ (**a**) and Δ (**b**) data fit at different angles of incidence.

According to the literature, FTO substrate is typically modeled as a multilayer structure. Typically, this structure is made up of approximately 340 nm of SnO_2_-F, followed by 25 nm of SiO_2_ and an additional 30 nm of SnO_2_, all supported by a 2 mm glass substrate [43]. Herein, a five-layer model was employed, which included a roughness layer, a bulk SnO_2_-F layer, a SiO_2_ layer, a transparent SnO_2_ layer and the glass substrate. The roughness layer was modeled as a combination of SnO_2_-F and voids (43%) using the Bruggeman effective medium approximation (EMA) [44], while the SnO_2_-F layer was described using the Lorentz oscillator model [44]. The optical constants for the glass substrate, SnO_2_, SiO_2_ and SnO_2_-F were obtained from the WVASE32 database. The model provides excellent data fittings with an MSE ~ 14. Figure 2a,b present both the simulated and measured data for the ψ and Δ spectra across different incident angles within the wavelength range of 300 to 900 nm for FTO substrate.

**Figure 3:**
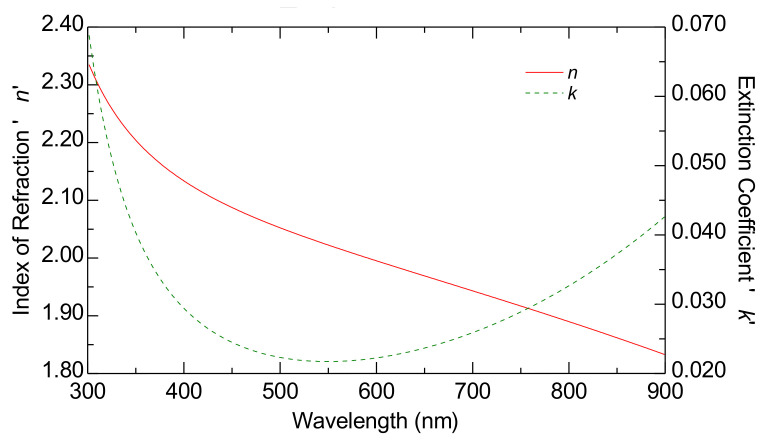
The dispersion laws of the FTO substrate estimated using ellipsometry characterization. The curves represent the index of refraction (red curve) and the extinction coefficient (green curve).

Table 1 presents the scheme of the optical model, while Figure 3 displays the estimated dispersion laws of the FTO substrate.

In the initial attempts to model TiO_2_-P25 films, it became evident that the films are nonhomogeneous, as their composition and optical properties vary with depth. As a result, single-layer models are inadequate for accurately representing these films. Therefore, it is necessary to employ models and methods capable of addressing this inhomogeneity. One effective approach is to use a graded layer model, which simulates a layer with depth-dependent inhomogeneity [40]. Two methods are commonly used to model nonhomogeneous layers. The first is the “Simple Graded Layer” method, where the layer is divided into several sub-layers (typically 5 to 15), each differing slightly from the previous one. In this model, the optical constants at the top of the film are a fraction of those at the bottom, with the variation progressing consistently between sub-layers. This change does not necessarily follow a linear pattern, but it remains regular throughout the structure. The second method, known as the “Function-Based Graded Layer,” is more complex. In this approach, the parameters within each sub-layer can vary independently, without requiring the same fractional change across all layers, allowing for more flexibility in the model. The “Function-Based Graded Layer” method was employed in this work. In contrast to the “Simple Graded Layer” method, which allows the optical constants at the top of the film to be slightly offset from those at the bottom while maintaining a similar overall profile, the “Function-Based Graded Layer” method permits variations in both the optical constants and the shape throughout the film. This approach provides greater flexibility, allowing for a more accurate representation of the film [40]. The optical properties of the TiO_2_-P25 films were initially investigated with General Oscillator (Gen OSC) [40], to fit the experimental data and extract the optical constants. The Gen OSC model was selected for its ability to represent the dielectric function as a sum of various oscillators, each corresponding to different physical processes.

The fitting process began with the selection of four Gaussian oscillators [40]. An iterative fitting approach was employed to refine the model parameters, minimizing the difference between the measured ellipsometric data and the values predicted by the model. This process involved adjusting the oscillator parameters until a satisfactory fit was achieved, as indicated by a low MSE.

The function-based graded layer model in WVASE32 was utilized to account for potential inhomogeneity within the material’s thickness [40]. Unlike the simple graded layer model [40], which only allows for a slight offset in the optical constants between the top and bottom of the film while maintaining a similar overall shape, the function-based graded layer model [40] permits variations in the shape of the optical constants profile throughout the depth of the film.

**Figure 4:**
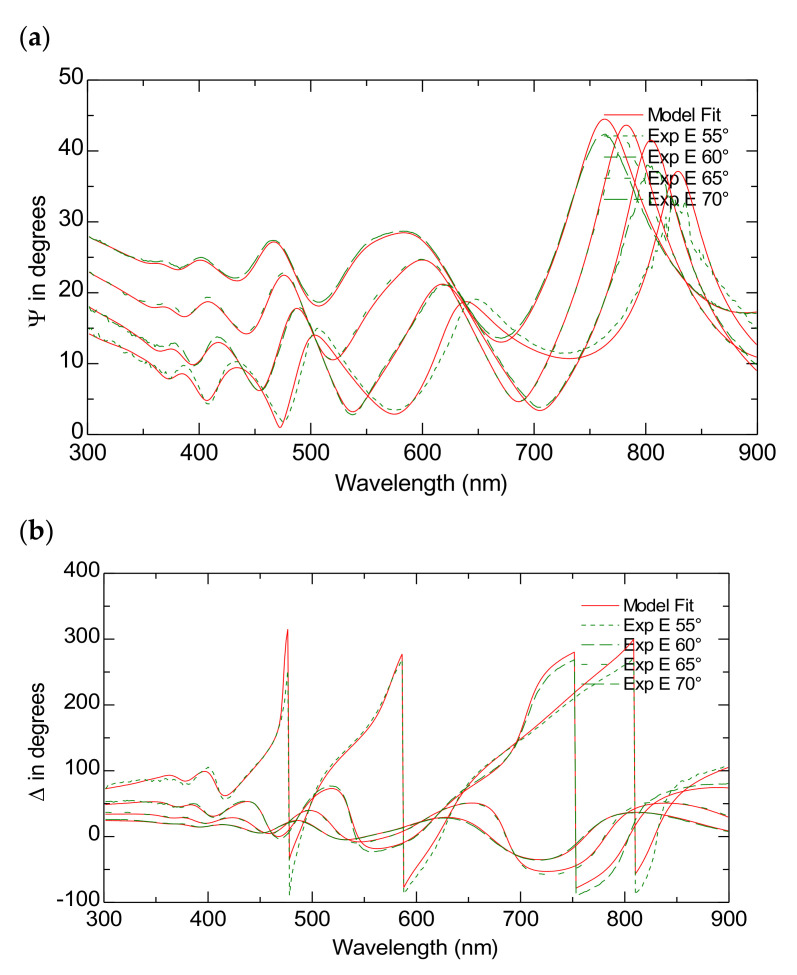
The Variable Angle Spectroscopic Ellipsometry measurements of the TiO_2_-P25 on FTO. The experimental and model-generated ψ (**a**) and Δ (**b**) data fit at different angles of incidence.

Figure 4a,b present both the simulated and measured data for the ψ and Δ spectra across various incident angles in the wavelength range of (300–900) nm for the TiO_2_-P25 films on FTO substrates. Table 2 presents the scheme of the model used to fit the experimental data presented in Figure 3. The model achieves data fittings with an MSE of approximately 13.

Table 2 shows the multilayer structure beginning with a 1 mm thick glass substrate (layer 0). Layers 1 through 4 form the FTO (fluorine-doped tin oxide) layers, as detailed in Table 1. Above these FTO layers, the structure progresses with layers 5 through 6, which include the more complex TiO_2_-P25 film. Notably, layer 5 is a substantial graded layer, 907.78 nm thick, modeled using a General Oscillator approach. This layer features a gradual change in its properties, such as the refractive index, throughout its thickness, which is crucial for finely tuning the optical response of the film. The topmost layer, layer 6, is 35.84 nm thick and modeled with the Effective Medium Approximation (EMA), incorporating 47.3% voids. This suggests a porous and less dense structure at the surface.

**Figure 5:**
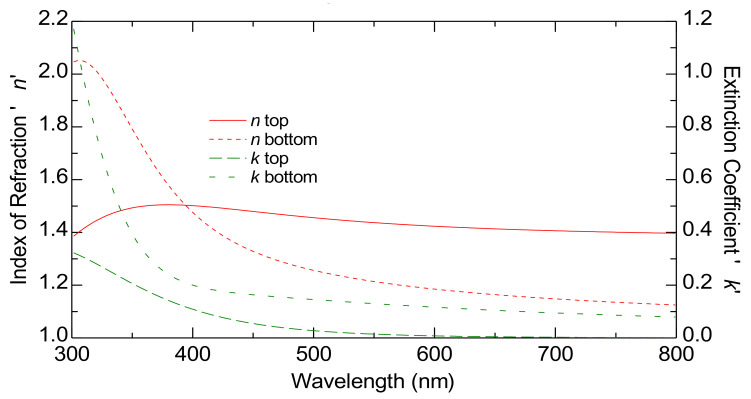
The graded optical constants at the bottom and top of the TiO_2_-P25 films on FTO substrate by ellipsometry characterization. The curves represent the index of refraction (red curves) and the extinction coefficient (green curves).

Figure 5 displays the estimated dispersion laws of TiO_2_-P25 films on an FTO substrate. This image highlights the complex interplay between the absorption and the refractive index within the layers, highlighting the depth-dependent optical properties that could be critical for applications requiring precise optical tuning, such as those involving photonic devices or advanced coatings.

In Figure 5, the solid red line shows *n* top while the dashed red line represents *n* bottom. Both refractive index *n* curves decrease with the increasing wavelength, which is normal for dielectric materials like TiO_2_. The extinction coefficients *k* are represented by dashed green lines, with *k* top as a long-dashed line and *k* bottom as a short-dashed line.

The higher *n* top compared to *n* bottom suggests that the region corresponding to *n* top has a higher density. This difference might indicate that the top part of the film is less porous or more compact compared to the bottom part. The model used in this study for 1000 nm thick films also works for thicker films, confirming its validity and reliability for greater thicknesses (see Supplementary Materials, Figures S1 and S2). This shows that the model is suitable for characterizing films with significant thickness.

**Figure 6:**
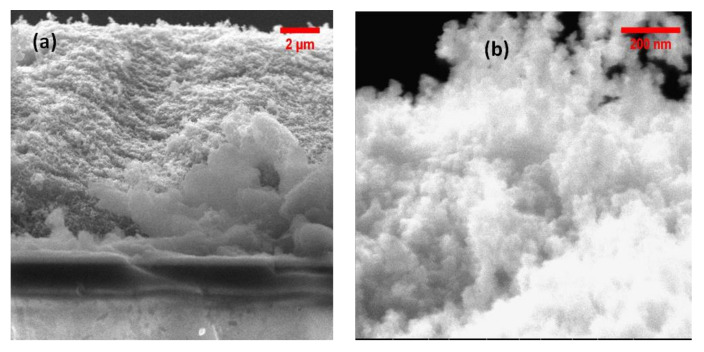
Scanning electron microscope images of the TiO_2_-P25 films (**a**) at lower and higher magnification (**b**) on FTO substrate.

Figure 6a,b show the porous structure of the films at different magnifications. TiO_2_ particles measure around 30 nm.

## 4. Conclusions

Herein, Variable Angle Spectroscopic Ellipsometry (VASE) was carried out to investigate the optical properties of thick films made of TiO_2_-P25, a material widely recognized for its photocatalytic effectiveness. The findings reveal that these films possess a highly porous and inhomogeneous structure, with significant variations in optical properties throughout their depth. Standard single-layer models proved inadequate in capturing these complexities, underscoring the necessity of employing a graded layer model for a more accurate representation of the films’ optical behavior. Therefore, a graded layer model was employed, which allowed for more accurate representation of the depth-dependent optical variations. This advanced modeling approach provided deeper insights into the internal structure of the films, particularly how the graded structural characteristics impact the overall optical behavior. The bottom layers of the films typically exhibited a significant increase in both refractive index and extinction coefficient, indicating greater density and higher absorbance compared to the top layers. Understanding these depth-dependent variations is essential for optimizing the use of TiO_2_-P25 films in technologies such as solar cells and optical devices, where precise control over material properties is critical.

## Figures and Tables

**Table 1:** A model of the FTO substrate.

**Layer Number**	**Layer Composition**	**Layer Thickness**
4	EMA	31.06 nm
3	SnO_2_-F	281.06 nm
2	SiO_2_	21.16 nm
1	SnO_2_	31.35 nm
0	glass	1 mm

**Table 2:** The Model of the TiO_2_-P25 films on FTO.

**Layer Number**	**Layer Composition**	**Layer Thickness**
6	EMA (GenOsc)/47.3% void	35.84 nm
5	Graded (GenOsc)	907.78 nm
4	EMA (SnO_2_-F)/43.2% void	31.06 nm
3	SnO_2_-F	281.06 nm
2	SiO_2_	21.16 nm
1	SnO_2_	31.35 nm
0	Glass	1 mm

## Data Availability

The data are contained inside the manuscript.

## Supplementary Materials

The following supporting information can be downloaded at: https://www.mdpi.com/article/10.3390/nano15020099/s1, Figure S1: Variable Angle Spectroscopic Ellipsometry measurements of 10.000-nm-thick TiO_2_-P25 films on FTO. Experimental and model generated ψ (a) and Δ (b) data fits at different angles of incidence; Table S1: Model of 10.000-nm-thick TiO_2_-P25 films on FTO; Figure S2: The optical constants at the bottom and top of the film of 10.000-nm-thick TiO_2_-P25 films on FTO substrate by ellipsometry characterization. The curves represent the index of refraction (red curves) and the extinction coefficient (green curves); Figure S3: Elemental composition of the films.
