# Supplementary material for: Optical Properties of Thick TiO2-P25 Films
Source: Nanomaterials (Basel). 2025 Jan 10;15(2):99. doi: 10.3390/nano15020099 (PMC11767985; doi:10.3390/nano15020099)
Supplement: Supplementary file 1 [file nanomaterials-15-00099-s001.zip › nanomaterials-3392383-supplementary.pdf]

Supplementary Materials

1) Theory of Ellipsometry

Ellipsometry is a nonperturbing optical technique that uses the change in the state of polarization of light upon reflection(or light transmission by a sample) for the characterization of surfaces, interfaces, and thin films.
Thin film thickness (t) and optical constants (n, k) are derived from the measurement.
Information is obtained from each layer interacting with the measured light beam.
Ellipsometry measures two values Δ and Ψ.
The two quantities (Δ and ψ) are measured as a function of wavelength and angle of incidence and are defined as follows:

$$tan\psi = \frac{|R^p|}{|R^s|} \qquad (1)$$

$$\Delta = \delta_p - \delta_s \qquad (2\ )$$

Ψ is thus the angle whose tangent gives the ratio of the amplitude attenuation (or magnification) upon reflection for the p and s polarizations, its value can be from zero to 90°. Δ, instead, gives the difference between the phase shifts experienced upon reflection by the p and s polarizations. The value of Δ can be between zero and 360°.
The terms *p* and *s* refer to the plane of incidence and the direction perpendicular to the plane of incidence.
The complex quantity ρ ,which is the complex ratio of the total reflection coefficients, is defined as:

$$\rho = \tan(\Psi)e^{i\Delta} = \frac{R_p}{R_s} \qquad (3)$$

where R_p and R_s contain information on the properties of the materials under investigation, where use of a capital R implies that the actual material structure can be multilayered and/or multicomponent. Analysis usually assumes flat parallel layers.
Rough surfaces and interfaces, as well as individual layers containing material mixtures can also be analyzed.

2) Data analysis

From the measurements in the ultraviolet /visible region, interband transitions (band structures) are characterized. In particular, the bandgap E_g can be deduced from the variation of α with $h\nu$. In the infrared region exists free carrier absorption induced by free electrons (or holes) in solids and electrical properties including carrier mobility, carrier concentration, and conductivity can be obtained. Moreover, in the infrared region, lattice vibration modes (LO and TO phonons) as well as local atomic structures can also be studied .
In the case of a bulk material we can provide the"pseudo" optical constants from the ellipsometry measurement:

$$\langle\tilde{\varepsilon}\rangle = sen^2(\phi)\left[1 + tan^2(\phi)\left(\frac{1-\rho}{1+\rho}\right)^2\right] \qquad (4)$$

This equation assumes there are no surface layers of any type. However, in any bulk material, there is typically a surface oxide or roughness, and the direct inversion would include these as part of the bulk optical constants.
For more complicated samples, which include several layers, inhomogeneity and surface roughness, the experimental ellipsometric angles collected at different incidence angles as a function of the energy of the impinging photons , must be compared with those provided by an appropriate model.
Figure 1 in the manuscript shows the data analysis process. The process begins with the execution of measurements. The model is then used to calculate the predicted determinations from Fresnel's equations, which describe each material's thickness and optical constants. In cases where these values are unknown, an estimate is used for preliminary calculations. The generated values (red lines in

Figure 1 in the manuscript) are then compared to the experimental data (green lines) using the mean square error (MSE) as a figure of merit to measure the fit quality .

$$MSE = \sqrt{\frac{1}{2N-M}\sum_{i=1}^{N}\left[\left(\frac{\left(\psi_i{}^{mod}-\psi_i{}^{exp}\right)}{\sigma_{\psi,i}{}^{exp}}\right)^2+\left(\frac{\left(\Delta_i{}^{mod}-\Delta_i{}^{exp}\right)}{\sigma_{\Delta,i}{}^{exp}}\right)^2\right]} \quad (5)$$

At the end of the process, we observe the resulting optical constants n and k, depicted in Figure 1 in the manuscript in blue and yellow lines. However, it's important to note that other quantities, such as surface roughness, can also be derived from ellipsometry.

The Mean Squared Error (MSE), as described in equation (5) quantifies the sum of squared deviations between the observed and predicted data points. Each deviation is scaled by the standard deviation of the respective measurement, ensuring that measurements with higher uncertainty (larger standard deviations) have a reduced influence on the overall fit. The optimization challenge is thus simplified to identifying the set of model parameters that produce a unique and absolute minimum for the MSE. This process involves minimizing the MSE value, which, for meaningful results, should be both small and exhibit a pronounced dependency on the chosen variable parameters. Importantly, the minimum MSE should represent the best possible alignment for the model, achievable only through a specific parameter set. Correlations between parameters or the inclusion of parameters with limited sensitivity can hinder the existence or identification of such a minimum [1].

Model of 10.000-nm-thick TiO_2-P25 films on FTO

Figure S1 (a) and Figure S1(b) present both the simulated and measured data for the ψ and Δ spectra across different incident angles within the wavelength range of 350 to 800 nm of 10.000-nm-thick TiO_2-P25 films on FTO substrates. Table SI presents the scheme of the model, while Figure S2 shows the estimated dispersion law. The model provides data fittings with a MSE of approximately 7.

(a)

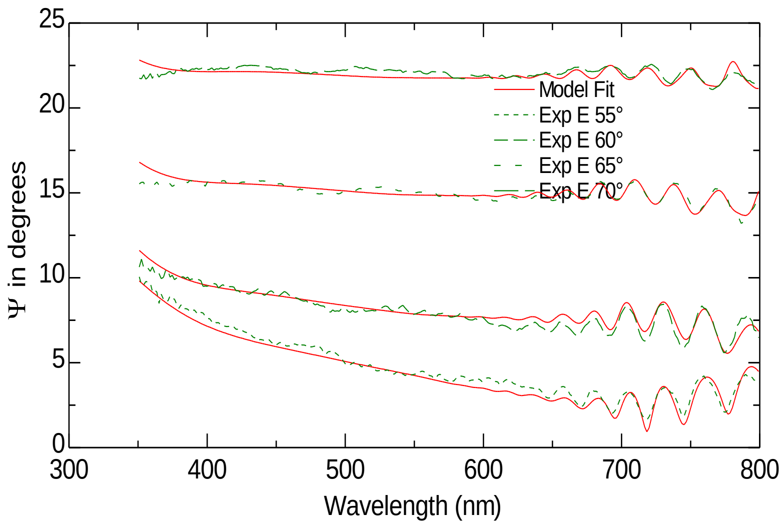

(b)

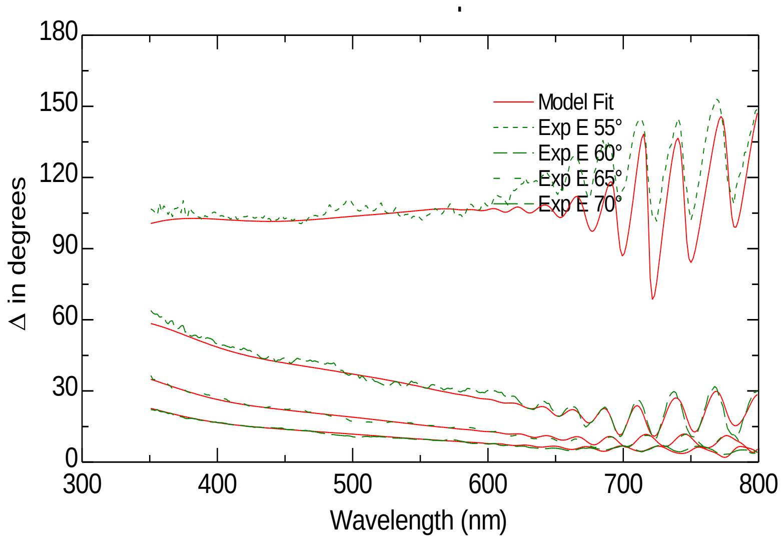

Figure S1: Variable Angle Spectroscopic Ellipsometry measurements of 10.000-nm-thick TiO_2-P25 films on FTO. Experimental and model generated ψ (a) and Δ (b) data fits at different angles of incidence.

Table SI: Model of 10.000-nm-thick TiO_2-P25 films on FTO.

Layer Number	Layer Composition	Layer Thickness
**6**	EMA (GenOsc)/50% void	32.04 nm
**5**	Graded (GenOsc)	10463.73 nm
**4**	EMA (SnO_2-F)/43.2% void	31.06 nm
**3**	SnO_2-F	281.06 nm
**2**	SiO_2	21.16 nm
**1**	SnO_2	31.35 nm
**0**	Glass	1 mm

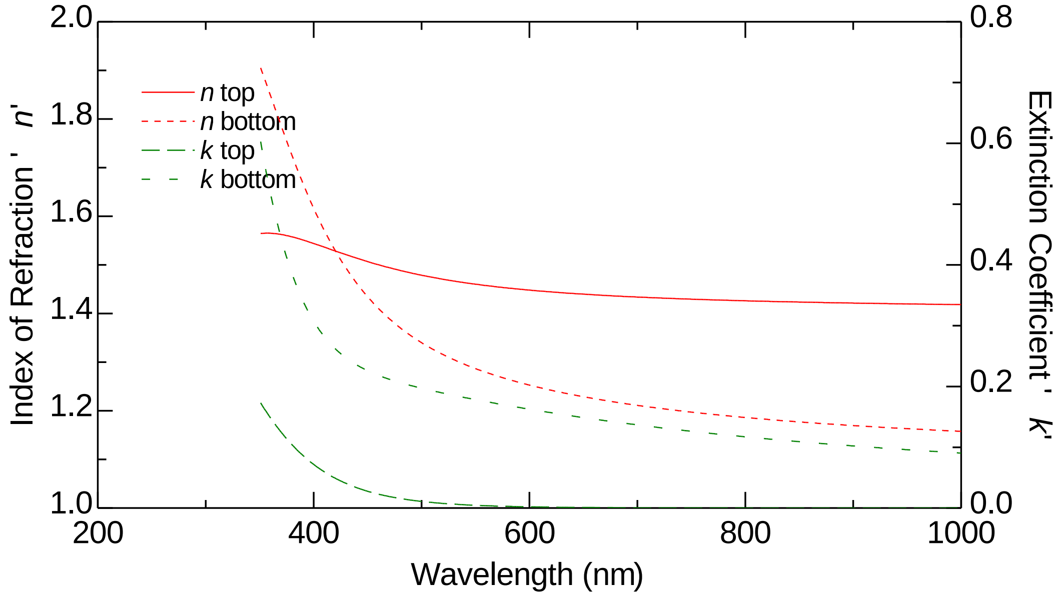

Figure S2. The optical constants at the bottom and top of the film of 10.000-nm-thick TiO_2-P25 films on FTO substrate by ellipsometry characterization. The curves represent the index of refraction (red curves) and the extinction coefficient (green curves).

3) EDS analysis of the films

In Figure S3 the Energy-dispersive X-ray spectroscopy (EDS) point elemental analysis in a representative point of the 10.000-nm-thick TiO_2-P25 films on FTO substrate is reported.
The EDS spectrum reveals the presence of Ti and O as the dominant elements, indicating the presence of a TiO_2 layer. The peaks for Ti and O are sharp and well-defined.The absence of Sn and Si signals in the EDX analysis of the TiO_2 sample suggests that the film was sufficiently thick to block contributions from the underlying FTO-coated glass substrate.

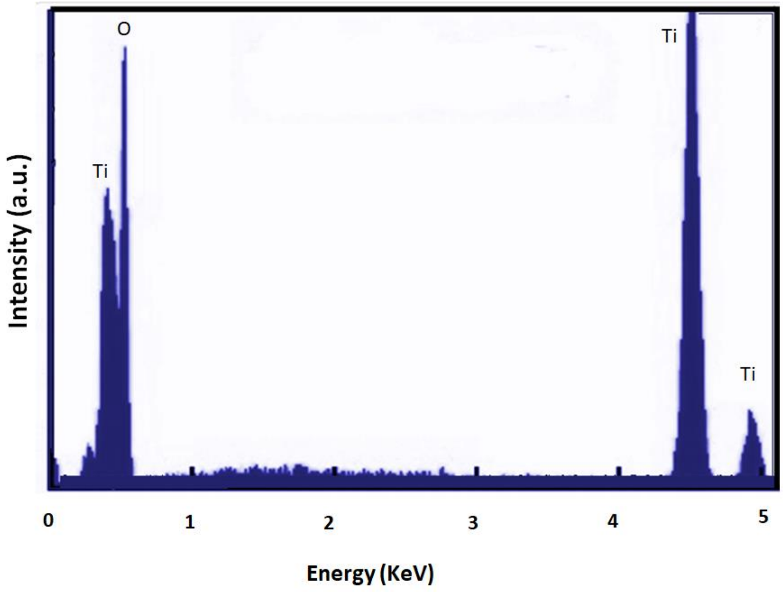

Figure S3: Elemental composition of the films.

1. J. A. Woollam Co. *WVASE manual "Guide to Using WVASE32";* 2010;
